# Genome-wide transcriptomics analysis identifies *sox7* and *sox18* as specifically regulated by gata4 in cardiomyogenesis

**DOI:** 10.1016/j.ydbio.2017.11.017

**Published:** 2018-02-01

**Authors:** Boni A. Afouda, Adam T. Lynch, Eduardo de Paiva Alves, Stefan Hoppler

**Affiliations:** a Institute of Medical Sciences, Foresterhill Health Campus, University of Aberdeen, Scotland, UK; b Centre for Genome-Enabled Biology and Medicine, King's College Campus, University of Aberdeen, Scotland, UK

**Keywords:** GATA factors, Sox factors, Heart muscle, Cardiomyogenesis, Embryonic stem cells, Xenopus

## Abstract

The transcription factors GATA4, GATA5 and GATA6 are important regulators of heart muscle differentiation (cardiomyogenesis), which function in a partially redundant manner. We identified genes specifically regulated by individual cardiogenic GATA factors in a genome-wide transcriptomics analysis. The genes regulated by *gata4* are particularly interesting because GATA4 is able to induce differentiation of beating cardiomyocytes in Xenopus and in mammalian systems. Among the specifically *gata4-*regulated transcripts we identified two SoxF family members, *sox7* and *sox18*. Experimental reinstatement of gata4 restores *sox7* and *sox18* expression, and loss of cardiomyocyte differentiation due to gata4 knockdown is partially restored by reinstating *sox7* or *sox18* expression, while (as previously reported) knockdown of *sox7* or *sox18* interferes with heart muscle formation. In order to test for conservation in mammalian cardiomyogenesis, we confirmed in mouse embryonic stem cells (ESCs) undergoing cardiomyogenesis that knockdown of *Gata4* leads to reduced *Sox7* (and *Sox18*) expression and that Gata4 is also uniquely capable of promptly inducing *Sox7* expression. Taken together, we identify an important and conserved gene regulatory axis from *gata4* to the SoxF paralogs *sox7* and *sox18* and further to heart muscle cell differentiation.

## Highlights

• Gata 4, 5 and 6 have redundant and non-redundant functions in heart development.
• RNA-seq analysis of Gata4, 5 and 6 knockdown experiments was carried out.
• Genes specifically regulated by Gata4, 5 and 6 were identified.
• The SoxF genes sox7 and sox18 were identified as specifically regulated by Gata4.
• Epistasis demonstrates a regulatory axis from Gata4 to Sox7/18 to cardiomyogenesis.

## Graphical abstract

**Figure:**
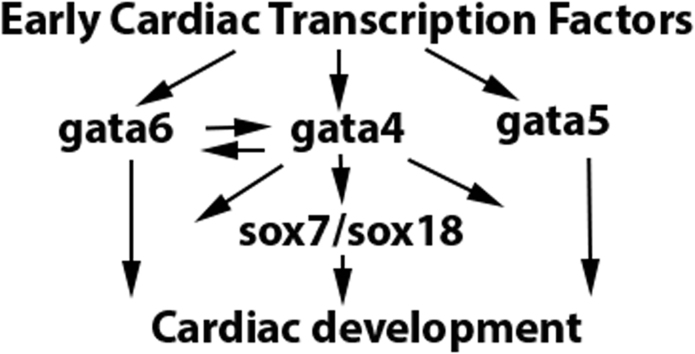

## 1 Introduction

Heart development and particularly heart muscle differentiation (cardiomyogenesis) is controlled by an intricate Gene Regulatory Network (GRN). This GRN involves prominent members of specific transcription factor gene families, such as the Nkx2, Mef2, Tbx and Gata gene families (Harvey and Rosenthal, 1999, Olson and Srivastava, 1996). Among these important transcription factors, GATA4, GATA5 and GATA6 are identified as the cardiogenic gata transcription factors (e.g. Peterkin et al., 2005). GATA4 is particularly important as a potent driver of cardiomyogenesis. In fact, carefully stage-controlled experimental activation of ectopically expressed GATA4 alone is sufficient to induce differentiation of functionally beating cardiomyocyte tissue from pluripotent stem-cell-like Xenopus animal cap explants (Afouda et al., 2008, Latinkic et al., 2003, see also below, e.g. Fig. 3). Experimentally forced expression of *Gata4* in mouse together with *Tbx5* and the chromatin remodelling protein *Baf60c* can reprogram somatic mesoderm into heart muscle cells (Takeuchi and Bruneau, 2009); and combined expression of *Gata4* with *Tbx5* and *Mef2c* was reported to convert cultured fibroblastic cells into a cardiac lineage (Ieda et al., 2010).

*Gata4* but also its paralogs *Gata5* and *Gata6* are required for normal heart formation in mammals (reviewed by Nemer, 2008) and human congenital cardiomyopathies are linked to mutations in the *GATA4* gene, including valve and septal defects (Garg et al., 2003, Rajagopal et al., 2007). However, *Gata4*, *Gata5* and *Gata6* have redundant functions as demonstrated by studies in mice embryos: *Gata4* and *Gata6* double mutants have complete acardia (Zhao et al., 2008), compound *Gata4/Gata5* mutants present severe cardiac defects (Singh et al., 2010) and compounds *Gata4/Gata5* as well as *Gata5/Gata6* mutants die embryonically or perinatally due to severe cardiac defects (Laforest and Nemer, 2011). Single Gata5 or single Gata6 both have milder phenotypes, though lack of *Gata5* in mice leads to bicuspid aortic valve formation (Laforest et al., 2011), asserting its importance for mammalian heart formation.

There is a significant degree of conservation of the molecular pathways involved in vertebrate heart formation; studies in zebrafish have identified that, in addition to cardia bifida, gata5 mutants (also known as faust) show loss of cardiomyocytes (Holtzinger and Evans, 2007, Reiter et al., 1999). Studies in both zebrafish and *Xenopus laevis* have confirmed that gata5 and gata6 have indeed redundant functions in cardiac progenitor specification (Haworth et al., 2008, Holtzinger and Evans, 2007, Peterkin et al., 2007).

Taken together, these observations confirm that the cardiogenic Gata factors represent genes with related function. In fact they derive through whole-genome duplications during the early vertebrate evolution (Dehal and Boore, 2005, Gillis et al., 2009, Ohno et al., 1968) from the same invertebrate ancestral gene (Ragkousi et al., 2011). This evolutionary origin reinforces the need to address questions about redundancy and unique functions of these cardiogenic gata transcription factors, since these genes may have partitioned an originally shared function (subfunctionalisation) or some of them may have acquired new specific functions (neofunctionalisation). Deciphering the genetic programme controlled by gata factors is therefore challenging, yet understanding the redundant and non-redundant specific function will be of great benefit for our understanding of heart development. We had previously uncovered some different requirements of cardiogenic gata factors for Xenopus cardiomyogenesis (Afouda and Hoppler, 2011). A more detailed understanding of the regulatory circuity controlled by individual cardiogenic gata factors will shed more light on the complex GRN that drive cardiomyogenesis.

Here, we sought to increase our knowledge of the roles of these factors in cardiomyogenesis (and consequently our knowledge of the cardiogenic GRN) by identifying their respective transcriptionally regulated genes on a genome-wide scale through RNA-seq analysis. We have used our established cardiogenic assay (Afouda, 2012, Afouda and Hoppler, 2009) of *Xenopus laevis* stem cell-like explants combined with gene knockdown to identify genes that are differentially affected by each of the cardiogenic gata genes. By taking advantage of the recent release of *X. laevis* genome assemblies and annotations (Xenbase.org: (Karpinka et al., 2015)) we have identified and then validated *sox7* and *sox18* as genes specifically regulated by *gata4* in cardiomyogenesis. Our genome-wide transcriptomics analysis therefore identifies within the GRN for cardiomyogenesis a conserved gene regulatory axis from *gata4* to the SoxF paralog genes *sox7* and *sox18* and further to heart muscle cell differentiation. Our identification of genes that are differently regulated by each of cardiogenic gata factors also provides a platform for future investigations that will further contribute to elucidating of molecular pathways and the GRN underpinning embryonic cardiomyogenesis.

## 2 Results

### 2.1 Xenopus stem-cell-like explants represent a reliable experimental model system for analysis of cardiogenic differentiation

**Fig. 1:**
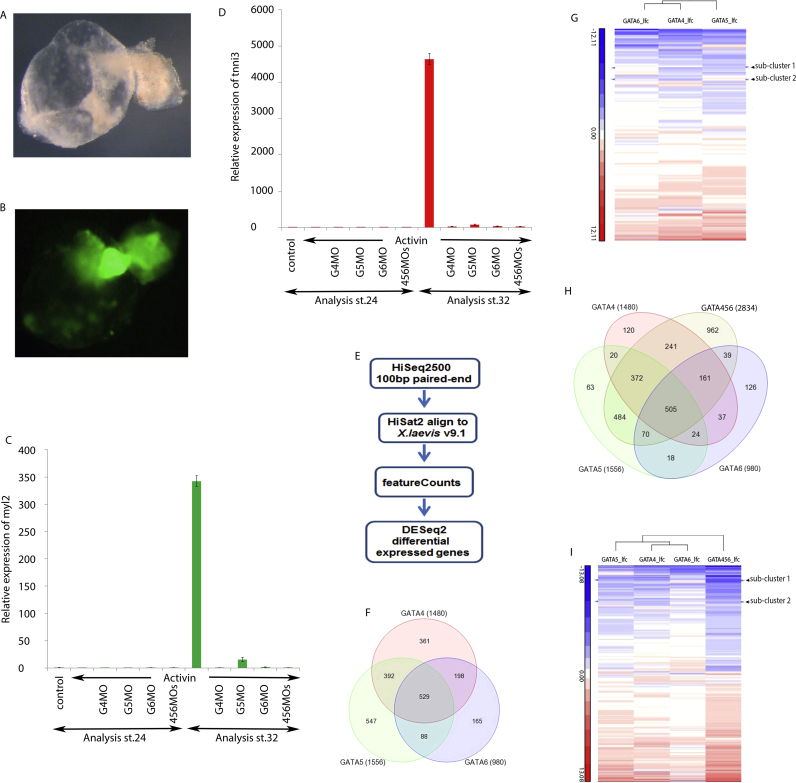
Activin-induced cardiac explants as cardiogenic assay to screen for genes specifically regulated by cardiogenic gata genes. Activin-injected animal caps from myl2-GFP reporter *Xenopus laevis* transgenic line were cultured until stage 45 and photographed in bright field (A) or with GFP filter (B) allowing visualisation of myl2-expressing cells throughout the beating explant. Total RNA was extracted from stage 32 wild-type *Xenopus laevis* explants injected with either Activin (Act) alone or together with gata4, 5 or 6 morpholinos (MOs) for real-time RT-PCR monitoring of myl2 (C) or tnni3 (D) expression. Following validation, samples were subjected to high-throughput sequencing where (E) represents schematic pipeline of RNA-seq bioinformatics analysis and (F, H) Venn diagrams of numbers of genes that are differentially expressed compared to Activin-injected control with at least a two-fold reduction in expression and an adjusted p value of<0.05. (G, I) Heatmaps of fold changes for differentially expressed genes (increased or decreased expression without any fold-change threshold applied) highlighting sub-clusters that are specifically regulated by gata4. Abbreviations: control, uninjected explant; G4MO, G5MO, G6MO and 456MOs representing respectively gata4, gata5, gata6 and all three combined morpholinos. Genes in sub-clusters 1 and 2 are listed in Fig. S1B, C.

Xenopus animal cap cells represent a pluripotent stem-cell-like tissue (Buitrago-Delgado et al., 2015) that can be induced to differentiate into various cell lineages by the addition of active inducer protein into the culture medium (Asashima et al., 2009) or by prior injection of inducer mRNA (Afouda, 2012, Warkman and Krieg, 2007, Weber et al., 2000). We have developed a reliable cardiomyogenesis assay consisting of injecting a low amount of Activin mRNA into animal cap embryonic explants (subsequently called cardiac explants, Afouda and Hoppler, 2009; Afouda et al., 2008). A myl2–green fluorescence (GFP) reporter line (myl2 is encoding myosin light chain 2, MLC2), which faithfully recapitulates the expression of this marker in cardiomyocytes (Latinkic et al., 2004) was used to show that explants from such embryos not only differentiate into rhythmically beating tissues and further allowed lineage tracing of cardiomyocytes and pan-myocardial expression of myl2 (Fig. 1A, B and suppl movie 1). The observed widespread expression of myl2 throughout each explant; and functional beating of essentially the entire explant implies that most of this tissue undergoes cardiac differentiation. This result demonstrates that our experimentally accessible cardiogenic model system is ideal for investigating the specific functions of cardiogenic gata factors in relative isolation from their other functions in other embryonic tissues. These advantages combined with the abundant amount of material available from each explant make this assay ideal for high-throughput sequencing approaches.

Supplementary material related to this article can be found online at doi:10.1016/j.ydbio.2017.11.017.

The following is the Supplementary material related to this article Movie 1.

Movie 1 *Beating tissue from Activin-induced cardiac explants.* Beating heart muscle tissue at control stage 45 derived from animal cap explant expressing activin β B from mRNA injected at one cell stage embryo. Note the rhythmic movement and the heart-like shape of the explant. One example shown from 45 cardiac explants used with n = 3 independent experiments.

### 2.2 Transcriptomics screen for gata4-, gata5- or gata6-regulated genes

We used this Xenopus stem-cell-like explant system to investigate at a genome-wide scale which genes are specifically regulated by different cardiogenic gata factors during cardiomyogenesis. Previously validated Morpholinos (MO) designed to knock down (inhibit) gata4, gata5, or gata6 expression were used (Afouda et al., 2005, Haworth et al., 2008, Peterkin et al., 2007). The efficacy of the gata MOs was further confirmed by observing altered inactive transcript isoforms in samples where splice MOs were injected (Fig. S1A). We prepared cultures of control cardiac explants, and cardiogenic explants with single gata4, gata5, or gata6 knockdown and with a triple gata4, 5, and 6 compound knockdown. We collected mRNA samples at stage 32 for subsequent RNA-seq analysis. Before sequencing, collected samples were validated for cardiac differentiation using quantitative RT-PCR (Taq man assay, Peterkin et al., 2003) by monitoring expression of terminal differentiation markers such as myl2 and tnni3 (encoding cardiac Troponin I, TnIc) (Fig. 1C, D). As expected, we observed reduced expression of these genes compared to control samples in single gata knockdowns, and a stronger reduction in the triple gata knockdown samples. Samples of mRNA from at least three validated independent biological experiments each were used for high-throughput sequencing (see M&M). Sequenced transcripts were identified by mapping to genes in the genome (*X. laevis* genome, version 9.1, Session et al., 2016), and counted to reveal expression differences between experimental samples [Fig. 1E and Materials and Methods; (Kim et al., 2015, Liao et al., 2014, Love et al., 2014)]. Threshold limits of two-fold difference in expression levels and statistical significance of 95% were chosen to assemble lists of differentially expressed genes (Fig. 1F, H; and Table 1 sheet 1 and Table 2 sheets 1 and 2 in Afouda et al., 2017). The chosen criteria were validated by confirmation that, as expected, expression of known cardiac differentiation markers, such as myl2 and tnni3, were identified as strongly reduced.

Using the above-mentioned bioinformatics strategy and criteria for the transcriptome-wide analysis to identify gata-dependent genes we found that expression of 1480, 1556 and 980 genes are decreased by either single *gata4*, *gata5* or *gata6* knockdown, respectively (Fig. 1F). Among these, 361 are specifically reduced by only the *gata4* knockdown, 547 specifically by the *gata5*, 165 specifically by the *gata6* single knockdown, while 529 genes are shared as they are reduced by either *gata4*, *gata5*, or *gata6* knockdown (Fig. 1F; and Table 1 in Afouda et al., 2017). Overall 2834 genes are affected by the triple *gata4*, *gata5* and *gata6* knockdown, of which 962 are only reduced in this triple knockdown (Table 2 in Afouda et al., 2017). This genome-wide analysis of the requirements for gene regulation of the cardiogenic gata transcription factor genes *gata4*, *gata5* and *gata6* confirms that they have shared, redundant but also gene-specific functions during cardiomyogenesis.

### 2.3 sox7 and sox18 are among genes specifically regulated by gata4 during early cardiomyogenesis

Because of the potency of gata4 to promote cardiomyogenesis (see above) we started here to focus on genes specifically affected by the *gata4* knockdown. We proceeded with analysing gene ontology (GO) terms associated with *gata4*-regulated genes (Fig. 1F; and see Afouda et al., 2017, Table 3 sheet 3 for all 1480 genes affected by gata4 knockdown and Table 3 sheet 4 for the 361 genes specially affected by the gata4 knockdown). Among the GO terms associated with genes requiring *gata4* functions are the GO terms “regulation of transcription” and “DNA binding” (see Table 3 in Afouda et al., 2017), suggesting that the *gata4* gene is required during cardiomyogenesis for regulating expression of other DNA-binding transcription factor genes. Among those specifically reduced by lack of *gata4* we discovered two paralogs of the SoxF family, *sox*7 and *sox18* (see Afouda et al., 2017, Table S3 sheets 1 and 2, highlighted in red). Interestingly, SoxF family members have been associated with cardiovascular development in different vertebrate species (reviewed by Francois et al., 2010), and in *Xenopus laevis sox7* and *sox18* are essential for heart development (Zhang et al., 2005b). We therefore decided to pursue this potential regulatory axis involving gata4 and SoxF genes in cardiomyogenesis. In order to test such a link further unsupervised clustering of our transcriptome data was carried out (Fig. 1G, I). Again *sox7* and *sox18* were contained in sub-clusters that are specifically affected by the *gata4* knockdown (Fig. 1G, I and Fig. S1B, C). Together, our transcriptomics analysis identify *sox7* and *sox18* as genes specifically regulated by *gata4* during early cardiomyogenesis.

### 2.4 Temporal progression from gata4 to sox7 and sox18 expression during cardiomyogenesis

**Fig. 2:**
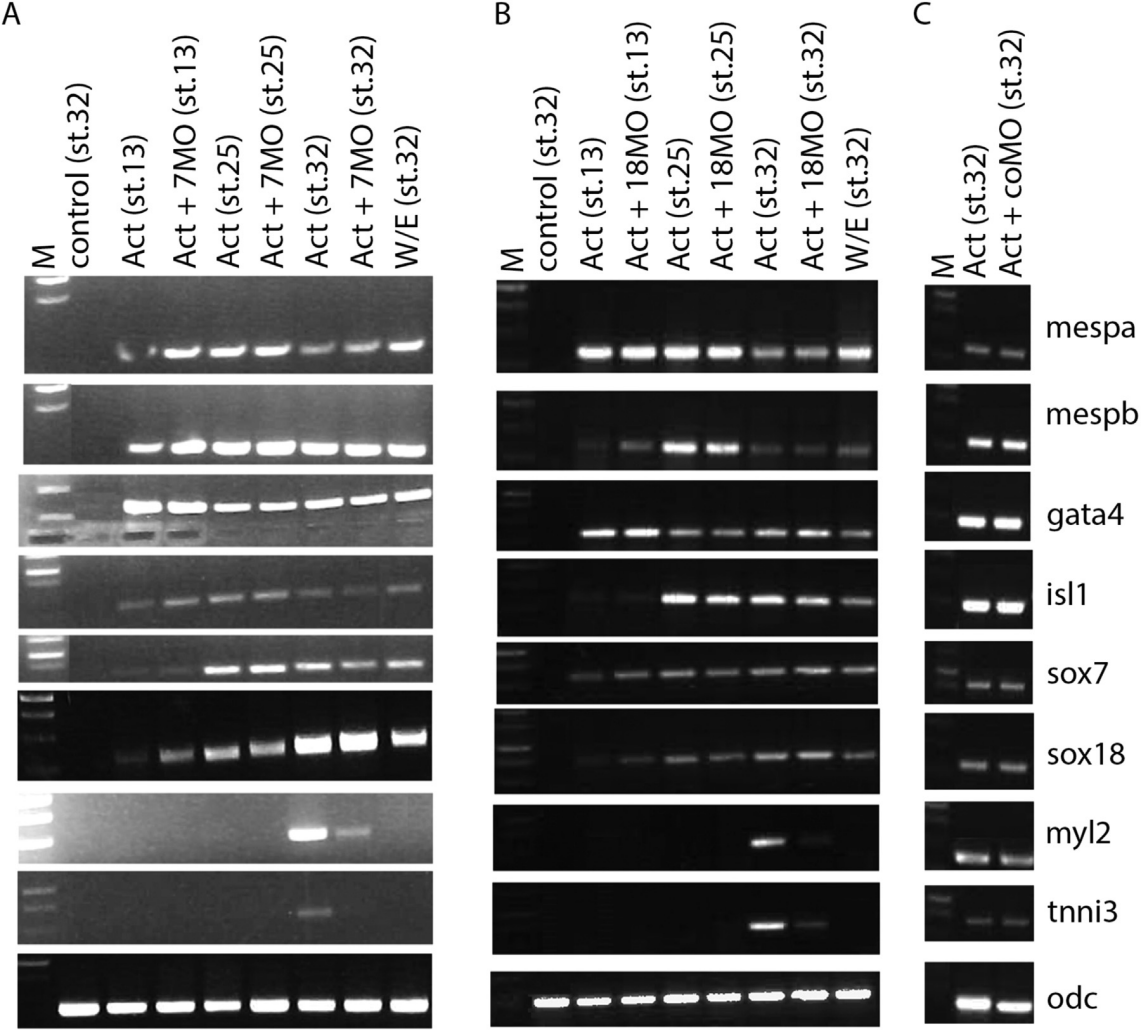
sox7 and sox18 are required for Activin-induced cardiac differentiation. Semi-quantitative RT-PCR assay monitoring cardiogenic gene expression (as indicated) in animal cap explants injected with Activin alone or in combination with sox7 and sox18 MOs, analysed at control stages 13, 25 and 32. Note that pronounced sox7 and sox18 expression starts after expression of cardiac progenitor markers mespa, mespb, and the early cardiac marker gata4. Note also that MO-mediated knockdown of sox7 or sox18 affect cardiac differentiation, as monitored by induction of cardiac differentiation markers myl2 and tnni3. M, Molecular size marker; control, uninjected explant; CoMO, control MO; 7MO, sox7 MO; 18MO, sox18MO; W/E, whole embryo; odc, ornithine decarboxylase (internal loading control).

The above data suggest that *sox7* and *sox18* expression in early cardiomyogenesis is downstream of *gata4* function and therefore predicted to be after *gata4* expression in development. Cardiogenic explants were collected at various time points of development and gene expression analysed by RT-PCR. In mouse, *Mesp1* has been identified as an early marker for cardiovascular progenitors and more importantly as a master regulator of cardiomyogenesis (Bondue and Blanpain, 2010, Bondue et al., 2008, David et al., 2008). In Xenopus, *mespa* has been shown to be the functional homologue of mammalian *Mesp1* (Kriegmair et al., 2013). We found *mespa* and its paralog *mespb* induced in cardiogenic explants from gastrulation onwards (Fig. 2). *gata4* as well as *isl1* (marker for undifferentiated but committed cardiac precursors in this context, Pandur et al., 2013) are induced early, mostly preceding expression of *sox7* and *sox18*, followed by expression of structural and differentiation markers *myl2* and *tnni3* (Fig. 2).

To test the requirement for *sox7* and *sox18*, we experimentally knocked down their expression with previously validated MOs (Zhang et al., 2005b). *sox7* or *sox18* knockdown caused no loss of early marker gene expression (*mespa*, *mespb*, *gata4* and *Isl1*) but had a strong effect later on expression of differentiation markers (*myl2* and *tnni3*) (Fig. 2). These observations further confirm that the explants recapitulate the cardiogenic development and place *sox7* and *sox18* temporally downstream of *gata4* and functionally upstream of cardiomyocyte differentiation.

### 2.5 sox7 and sox18 are required downstream in gata4-induced cardiomyogenesis

**Fig. 3:**
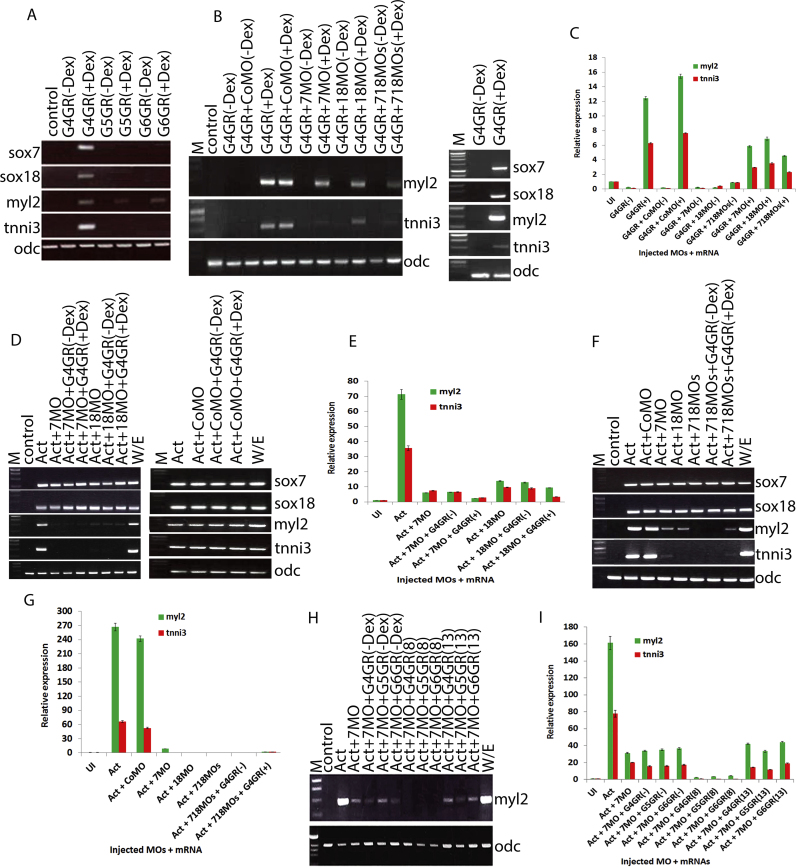
sox7 and sox18 are required downstream of gata4 for cardiac differentiation. Semi-quantitative (A, B, D, F and H) and quantitative RT-PCR (C, E, G and I) assay monitoring cardiac differentiation marker gene expression in control stage 32 explants. Explants injected with 1000 pg of mRNA encoding dexamethasone-inducible gata4 (G4GR), gata5 (G5GR) and gata6 (G6GR) (A, B and C); or Activin-induced cardiac explants in the presence of control, sox7, sox18 or both SoxF MOs with mRNA encoding dexamethasone-inducible gata4, 5, 6 (D to I). Note that gata4, 5 and 6 induce cardiac differentiation to a different extent (A). Note also that both sox7 and sox18 MOs result in reduced induction of cardiac differentiation markers (myl2, tnni3) (B to I and that this cannot be recovered by overexpression of either gata4 or the other paralogs gata5 or 6 (D to I)). Note further that simultaneous knock down of sox7 and sox18 essentially abolishes expression of cardiac differentiation markers (B, C, F and G) and that neither sox7 nor sox18 is required for their own or each other's expression (D, F). CoMO, control MO; 718MOs, sox7 and sox18 MOs; Dex, Dexamethasone added at control stage 8 apart H and I as indicated at stages 8 and 13. Other abbreviations are as in Fig. 2.

In order to test for the ability of gata4 to induce *sox7* and *sox18* expression during cardiomyogenesis we used the previously established cardiogenic assay in animal cap explants driven by a hormone inducible gata4 (Afouda et al., 2008, Latinkic et al., 2003). All three cardiogenic gata factors are capable of inducing heart differentiation marker gene expression in this assay, but clearly to different extents, with only gata4 being able to drive differentiation into functionally beating cardiomyocytes (Afouda and Hoppler, 2011). We found that these differences are correlated with our finding that only gata4 is able to induce *sox7* and *sox18* expression in this assay (Fig. 3A). This result suggests that induction of beating cardiomyocytes by gata4 might involve these two paralogs of the SoxF family.

We therefore addressed the question whether these SoxF genes are required for gata4 to induce functional cardiomyocyte differentiation (see also (Zhang et al., 2005b)). To this end, we knocked down *sox7* and *sox18* in gata4-induced cardiomyogenesis and monitored expression of the cardiac differentiation markers *myl2* and *tnni3* (Fig. 3B, C). *sox7* or *sox18* knockdown causes substantially reduced cardiac differentiation as monitored by reduced expression of marker genes (Fig. 3B, C). Importantly we had made certain that the exogenously over-expressed protein is efficiently synthesised in the presence of the MOs providing evidence that the observed effect is due to the intrinsic activity of GATA4 and its regulatory relationship with *sox7* and *sox18* (Fig. S1D). Our results show that *sox7* and *sox18* function is required downstream of gata4 for induction of functional cardiomyocyte differentiation.

In our effort to establish the epistatic functional relationship of *gata4* with *sox7* and *sox18* during cardiac differentiation we next reinstated gata factor gene expression in a temporally controlled manner in Activin-induced cardiac explants in which *sox7* and *sox18* function was knocked down. To this end we used the same hormone-inducible version of gata4 (as introduced above and previously described in Afouda et al., 2005; Afouda and Hoppler, 2011; Afouda et al., 2008).

Induction of cardiomyogenesis was severely affected by knockdown of either *sox7* or *sox18*, as measured by expression of terminal differentiation markers *myl2* and *tnni3* (Fig. 3D, E); and cardiomyogenesis could not be restored by experimentally over-activating gata4 function. Our data confirm the requirements for both paralogs of SoxF family downstream of gata4 function during cardiomyocyte differentiation, which raises the question about possible redundancy between these two factors.

To examine redundancy between *sox7* and *sox18* function, we simultaneously knocked down both in cardiogenic explants and proceeded with monitoring expression of cardiac differentiation markers (Fig. 3F, G), as well as functional differentiation (suppl movie 2 and Fig. S1E). Re-instating gata4 activity was unable to substantially recover expression of marker genes for cardiogenic differentiation (Fig. 3F, G). It is worth noting that neither *sox7* nor *sox18* knockdown affects their own or each other's expression. Additionally, reduced cardiac differentiation in the *sox7* knockdown (or *sox18* knockdown, data not shown) can also not be recovered by overexpression of either gata5 or gata6 (Fig. 3H, I), confirming that neither gata5 nor gata6 function downstream of sox7 (or sox18) function. As the amounts of the gata proteins present after activation are comparable (Fig. S1F), we can be certain that the observed effects are due to the intrinsic potential of each of these GATA factors in these experimental assays.

Supplementary material related to this article can be found online at doi:10.1016/j.ydbio.2017.11.017.

The following is the Supplementary material related to this article Movie 2, Movie 3, Movie 4, Movie 5, Movie 6, Movie 7.

Movie 2 *cardiac explants (uninjected control for sox7 and 18 morpholino experiment).* Control tissue at control stage 45 derived from uninjected animal cap explants. Note absence of any rhythmic movement or heart-like shape of the explant (see numerical analysis presented in Fig. S1E). Examples shown from 50 cardiac explants used each with n = 3 independent experiments.

Movie 3 *Activin-induced cardiac explants (no morpholino control for sox7 and 18 morpholino experiment).* Beating heart muscle tissue at control stage 45 derived from animal cap explant expressing activin β B from mRNA injected at one cell stage embryo without sox7 or sox18 MOs. Note the rhythmic movement and the heart-like shape of the explant (see numerical analysis presented in Fig. S1E). Examples shown from 52 cardiac explants used each with n = 3 independent experiments.

Movie 4 *Activin-induced cardiac explants (CoMO control for sox7 and 18 morpholino experiment).* Beating heart muscle tissue at control stage 45 derived from animal cap explant expressing activin β B from mRNA injected at one cell stage embryo together with control MO. Note the rhythmic movement and the heart-like shape of the explant (see numerical analysis in Fig. S1E). Examples shown from 48 cardiac explants used each with n = 3 independent experiments.

Movie 5 *Activin-induced cardiac explants in the presence of sox7 morpholino.* Reduced beating of heart muscle tissue at control stage 45 derived from animal cap explant expressing activin β B from mRNA injected at one cell stage embryo together with sox7 MO. Note much reduced rhythmic movement and the heart-like shape of the explant in the presence of the sox7 MO (see numerical analysis in Fig. S1E). Examples shown from 40 cardiac explants used each with n = 3 independent experiments.

Movie 6 *Activin-induced cardiac explants in the presence of sox18 morpholino.* Reduced beating of heart muscle tissue at control stage 45 derived from animal cap explant expressing activin β B from mRNA injected at one cell stage embryo together with sox18 MO. Note much reduced rhythmic movement and the heart-like shape of the explant in the presence of the sox18 MO (see numerical analysis in Fig. S1E). Examples shown from 42 cardiac explants used each with n = 3 independent experiments.

Movie 7 *Activin-induced cardiac explants in the presence of sox7 and sox18 morpholino.* Reduced beating of heart muscle tissue at control stage 45 derived from animal cap explant expressing activin β B from mRNA injected at one cell stage embryo together with sox7 and sox18 MOs. Note much reduced rhythmic movement and the heart-like shape of the explant in the presence of the sox7 and sox18 MOs (see numerical analysis in Fig. S1E). Examples shown from 40 cardiac explants used each with n = 3 independent experiments.

### 2.6 sox7 and sox18 can partially substitute for gata4 function during cardiomyogenesis

**Fig. 4:**
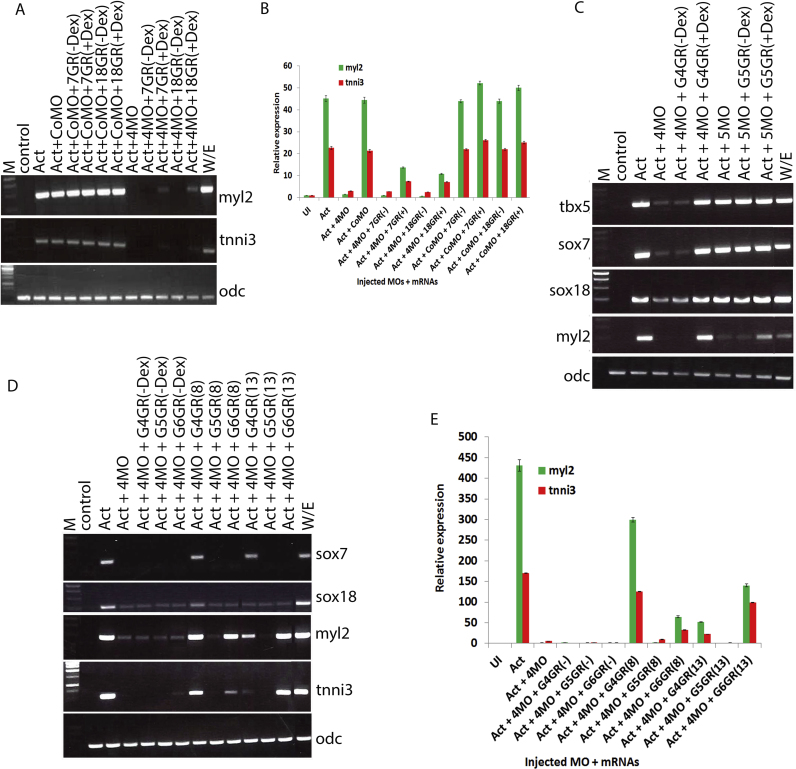
sox7 and sox18 are essential downstream mediators of gata4 during cardiomyogenesis. Activin-induced cardiac explants injected with control, gata4 or gata5 MOs together with mRNA encoding inducible sox7 (7GR), sox18 (18GR) or gata proteins (G4GR, G5GR and G6GR), as indicated, which were activated before gastrulation (8, stage 8) (A to C) and during late gastrula (13, stage 13), as indicated (D and E). Semi-quantitative (A, C and D) and quantitative (B and E) RT-PCR assay monitoring indicated markers analysed at control stage 32. Note the partial recovery (A, B) of the effect of gata4 inhibition on cardiac differentiation by experimentally activated SoxF proteins (7GR and 18GR). Note also that only gata4 knock down affects sox7 and sox18 expression and that only gata4 when reinstated can recover sox7 and sox18 expression. Note further the stage-dependent different intrinsic cardiac inducing properties of the three cardiogenic gata paralogs (as revealed by expression of cardiac differentiation markers myl2 and tnni3). Abbreviations are same as in previous figures. 200 pg and 1000 pg of mRNA were injected for overexpression of sox and gata proteins respectively.

We wondered to what extent SoxF function was able to mediate *gata4* function in cardiomyogenesis. In order to address this question we developed an experimental design that would allow us to re-instate sox7 or sox18 activity in a temporally controlled manner in a gata4 knockdown background. To this end we fused the sox7 and the sox18 coding sequence in frame with the ligand-binding domain of the human glucocorticoid receptor (GR) (see Materials and Methods for details). The obtained fusion construct called respectively sox7GR and sox18GR were tested in animal cap explants and found to induce expression of terminal cardiac differentiation markers myl2 and tnni3 (data not shown). Inhibition of gata4, as expected caused a clear reduction of cardiogenic marker gene expression (cf. Afouda et al., 2008); interestingly, marker gene expression is noticeably reinstated to some extent when either sox7 or sox18 is experimentally activated (Fig. 4A, B). As the efficiencies of the over-expressed sox7 or sox18 proteins are not affected by the MOs (Fig. S1G), we can conclude that sox7 or sox18 is able to induce expression of cardiac differentiation markers when there is insufficient *gata4* function and that *sox7* and *sox18* are therefore able to mediate some *gata4* function during cardiac differentiation.

### 2.7 The gata4-soxF regulatory axis mediates non-redundant functions of cardiogenic gata factors in early versus later cardiomyogenesis

The aim of this investigation was to identify specific functions for the cardiogenic gata genes in the GRN driving vertebrate cardiomyogenesis. We had previously discovered non-redundant functions for gata4 and gata5 (Afouda and Hoppler, 2011), briefly with gata4 required for earlier and gata5 for later stages of cardiomyogenesis. Our transcriptomics analysis here has now identified the SoxF genes sox7 and sox18 as specifically regulated by gata4 (Fig. 1) and the above experiments confirm a functional role for sox7 and sox18 downstream of gata4 during cardiomyogenesis (Fig. 2, Fig. 3, Fig. 4A, B). We therefore wondered to what extent sox7 and sox18 function fits with this concept of early versus late functions of cardiogenic gata factors during cardiomyogenesis and therefore particularly focussed here on gata4 and gata5. In addition, to avoid the supplied exogenous GATA mRNAs being targeted by the MOs we have here used the previously validated splice MOs to strictly target endogenous gata4 and gata5 (Afouda and Hoppler, 2011, Haworth et al., 2008).

In the gata4 knockdown, as expected we observe strongly reduced expression of early cardiac markers such as *tbx5* and of both sox7 and sox18; and —presumably as a consequence— of cardiac differentiation markers such as *myl2*; all of which could be fully recovered with experimentally reinstated *gata4* function (Fig. 4C). In contrast inhibition of *gata5*, as expected only affects the later stage differentiation marker *myl2* but neither *sox7* nor *sox18* expression; nor early stage cardiac markers (Fig. 4C). Interestingly reinstating gata5 activity could substantially reinstate the expression of the differentiation marker *myl2*. These observations confirm that gata5 function during cardiomyogenesis is *sox7*- and *sox18*-independent, in contrast to its paralog *gata4*.

We then decided to explore to what extent the other cardiogenic gata factors gata5 and gata6 could replace *gata4* in the regulation of *sox7* and *sox18* during cardiomyogenesis. We conducted experiments in which *gata4* function was knocked down while concomitantly re-instating gata4; or activating overexpressed gata5 or gata6, instead. We discovered that in such an experimental setting, reduced expression of *sox7* and *sox18* as well as of the cardiac differentiation markers *myl2* and *tnni3* could be recovered, as expected, when gata4 was reinstated, either at stage 8 or stage 13; but experimentally activated gata5 or gata6 could only recover expression of differentiation markers but neither sox7 nor sox18 (Fig. 4D, E). Our data show that only gata4 regulates *sox7* and *sox18* during cardiomyogenesis and that the other cardiogenic gata factors gata5 and gata6 cannot compensate for *gata4* in this assay and must therefore here function via *sox7*- and *sox18*-independent mechanisms.

### 2.8 sox7 and sox18 are required for heart muscle differentiation in the embryo

**Fig. 5:**
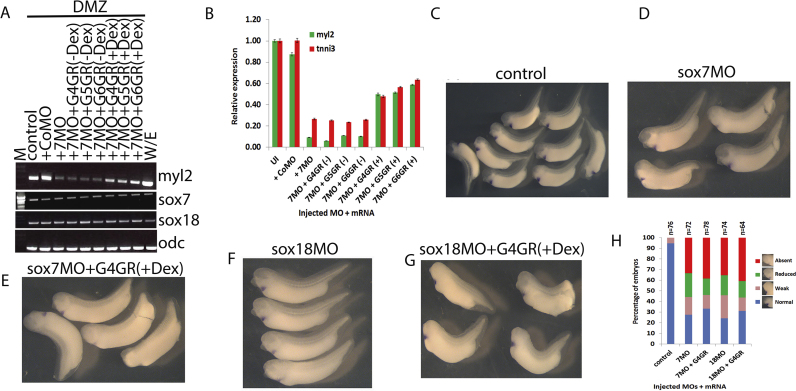
sox7 and sox18 are required for endogenous cardiac differentiation in vivo. Semi-quantitative (A) and quantitative (B) RT-PCR analyses of cardiac differentiation (*myl2* expression) in Xenopus stage 32 dorsal marginal zone (DMZ) explants injected with sox7 MO together with 200 pg of mRNAs encoding dexamethasone-inducible gata proteins, as indicated. Note considerable requirement of *sox7* function for cardiac differentiation (myl2 expression) and the ability of experimentally activated gata4, 5 or 6 to cause only a partial recovery of cardiac differentiation in a sox7 knockdown (compare with Activin-induced cardiomyogenesis, see Fig. 3H and I). (C - G) Whole-mount in situ hybridisation analysis of tnni3 expression of stage 32 embryos which are either uninjected (C) or had been injected at the four-cell stage in the two dorsal blastomeres with sox7 MO (D), sox7 MO plus gata4GR mRNA (E), sox18 MO (F) or sox18 MO plus gata4GR mRNA (G). Dexamethasone was added at stage13 to activate the inducible gata4 protein (in E and G). Note that embryos with depleted sox7 and sox18 have reduced tnni3 expression compared to wild type (C) ranging from weak to complete reduction (D and F), which cannot be recovered with activated gata4 proteins (E and G). (H) Quantified data of tnni3 expression in each condition.

Activin-induced cardiac explants are good for studying cardiomyogenesis in relative isolation from neighbouring embryonic tissues and therefore for studying cardiogenic gata and SoxF function specifically in cardiogenic mesoderm. Dorsal marginal zone (DMZ) explants differentiate into heart tissue in the absence of added factors (Foley and Mercola, 2005, Schneider and Mercola, 2001), presumably because the cardiac precursors reside within this part of intact gastrulating Xenopus embryos (Afouda and Hoppler, 2009). These DMZ explants therefore represents a first step in putting the cardiogenic tissue back into the normal wider embryonic context. We conducted similar experiments using DMZs to those described above with Activin-induced cardiac explants (cf. Fig. 3H). DMZs in which *sox7* is knocked down (or *sox18*, data not shown) have reduced cardiac differentiation marker gene expression as measured by semi-quantitative and quantitative RT-PCR (Fig. 5A, B). Interestingly when any one of the cardiogenic gata factors is activated we observe a noticeable recovery of cardiomyogenesis, which is different from what had been observed above in Activin-induced cardiogenic explants (cf. Fig. 3H). This observation suggests existence of endogenous tissues or factor(s) in DMZ explants that are not present within Activin-induced cardiac explants, which allow the paralogs gata5 and 6 to reinstate cardiac differentiation even in a *sox7* (or *sox18*) knock down.

As a further step for putting the cardiogenic tissue back into the normal embryonic context we studied a whole embryo *sox7* knockdown or *sox18* knockdown by targeting injection of sox7 or sox18 MOs into the prospective heart tissue of 4-cell stage Xenopus embryos (Afouda et al., 2008, Foley and Mercola, 2005, Martin et al., 2010, Nascone and Mercola, 1995, Sater and Jacobson, 1990). Although mixed phenotypes were observed, there is a reduction of expression of the myocardium differentiation marker *tnni3* in the *sox7* or the *sox18* knockdown (Fig. 5C-H). Additionally we found no difference between those knockdown embryos and knockdown embryos in which gata4 activity has been experimentally activated (Fig. 5E, G). We therefore conclude that sox7 and sox18 are required for normal myocardium differentiation in a manner consistent with functioning downstream of gata4 during cardiac differentiation.

### 2.9 Sox7 regulation by Gata4 is conserved in mammalian cardiomyogenesis

**Fig. 6:**
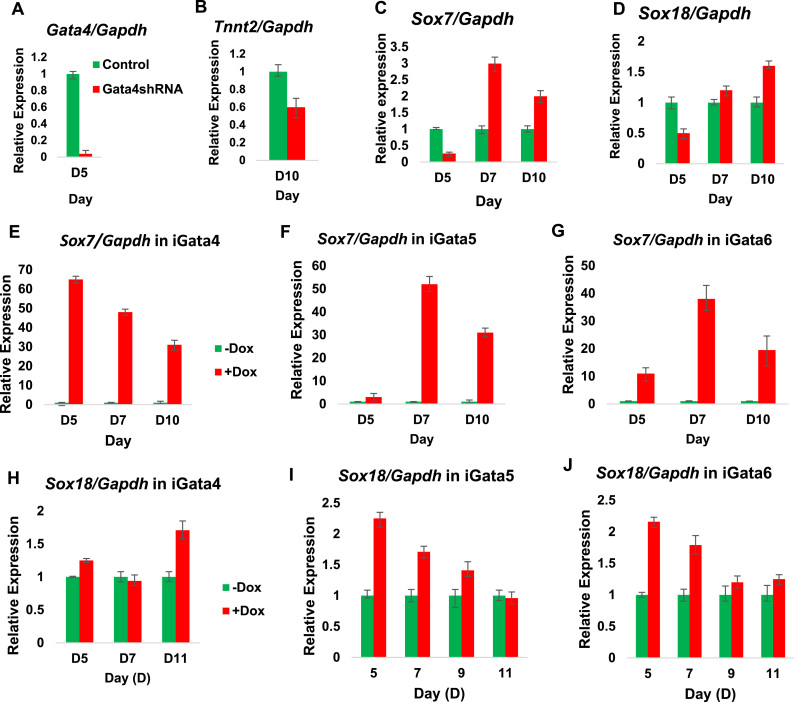
Sox7 is required during mouse ES cell differentiation into cardiomyocytes (A) RT-qPCR analysis of *Gata4* expression at day 5 (D5) of cardiomyocyte differentiation. *Gata4* expression was significantly decreased in cells transfected with *Gata4* shRNA compared to control cells. (B) *Tnnt2* expression was decreased at D10 in *Gata4* knockdown cells compared to untransfected controls. (C) *Sox7* expression during cardiomyocyte differentiation. At D5, *Sox7* expression was reduced in cells transfected with *Gata4* shRNA compared to controls, though at subsequent stages expression was increased. (D) *Sox18* expression was also reduced at D5 in cells transfected with *Gata4* shRNA compared to untransfected controls, whereas expression was increased during later stages of differentiation. (E) RT-qPCR analysis of *Sox7* expression in inducible *Gata4* (iGata4) mouse ES cells. Following doxycycline (+dox) induction at D4, *Sox7* was rapidly upregulated at D5 compared to control (-dox). (F and G) RT-qPCR analysis of *Sox7* expression in iGata5 and iGata6 cells. *Sox7* expression was only moderately increased at D5 in induced cells compared to iGata4 (E). *Sox7* expression was later increased at D7 and D10 compared to controls. (H) RT-qPCR analysis of *Sox18* in iGata4 cells. *Sox18* expression was only slightly increased at D5 following doxycycline induction at D4, compared to uninduced controls. (I and J) RT-qPCR analysis of *Sox18* expression in iGata5 and iGata6 cells. *Sox18* expression was increased at D5 following doxycycline induction at D4 in iGata5 and iGata6 cells, compared to uninduced controls.

Molecular pathways involved in cardiomyogenesis are generally well conserved among vertebrates (Bruneau, 2002, Zaffran and Frasch, 2002). In order to test for conservation in mammalian cardiomyogenesis of the proposed *Gata4* to *Sox7/Sox18* regulatory axis we used mouse Embryonic Stem Cells (mESCs) cultured as Embryonic Bodies (EBs) that spontaneously undergo cardiomyocyte differentiation (Boheler et al., 2002). We confirmed that *Gata4* (Fig. 6A), the cardiomyocyte differentiation marker *Tnnt2* (Fig. 6B), *Sox7* (Fig. 6C) and *Sox18* (Fig. 6D) are expressed in these EBs. In a Gata4 shRNA knockdown (Fig. 6A-D), expression of *Sox7* and *Sox18* are reduced (Fig. 6C, D), as is the expression of *Tnnt2* (Fig. 6B). This confirmed the *Gata4* requirement for normal *Sox7* and *Sox18* expression during cardiomyocyte differentiation. In order to test for the ability of cardiogenic Gata factors to regulate *Sox7* and *Sox18* expression, we used mESC lines in which the expression of individual cardiogenic gata factors can be experimentally activated (iGata4, iGata5, iGata6, Turbendian et al., 2013). Experimental activation of *Gata4* expression prompted a quick and dramatic induction of Sox7 expression (Fig. 6E). Other cardiogenic Gata factors were only able to cause a much more modest initial induction of *Sox7* expression (iGata6, Fig. 6G) or only a delayed response (iGata5 and iGata6, Fig. 6F, G), suggesting indirect mechanisms. In contrast, experimental overexpression of *Gata4* caused only a slight increase in *Sox18* expression (Fig. 6H), whereas ectopic expression of *Gata5* and *Gata6* cause a greater increase in *Sox18* expression (Fig. 6I, J). These findings suggest that regulation by Gata4 of the SoxF genes *Sox7* and *Sox18* is conserved in the context of mammalian cardiomyogenesis. However, in contrast to animal caps, we find that *Sox18* is regulated by all three cardiogenic Gata factors in EBs (Fig. 6I-J).

## 3 Discussion

Wnt/β-catenin signalling can inhibit early cardiomyogenesis (e.g. Ueno et al., 2007), which we discovered is mediated by negative regulation of gata gene expression (Afouda and Hoppler, 2009, Afouda et al., 2008, Martin et al., 2010). Our subsequent studies had additionally discovered some specific functional requirements of different cardiogenic gata factor genes for cardiomyogenesis (Afouda and Hoppler, 2011). In this study we aimed to explore such gata gene-specific functions more comprehensively using genome-wide transcriptomics analysis, in order to widen our general understanding of the gene regulatory network (GRN) directing cardiomyogenesis. Using gene knockdown approaches we have identified genes that are specifically regulated by individual cardiogenic gata factor genes *gata4*, *gata5* or *gata6*; as well as those regulated by either one of them, and those regulated by all three of them together.

We are here focussing only on some aspects of the wealth of information that was generated in our high-throughput sequencing experiments, especially on genes specifically regulated by *gata4*. Gene ontology (GO) analysis suggests that *gata4* tends to regulate other transcription factor genes during cardiomyogenesis, among which we identified the SoxF subfamily genes *sox7* and *sox18* (Fig. 1). This requirement of specifically *gata4* for *sox7* and *sox18* expression is confirmed in subsequent validation experiments (e.g. Fig. 4), which also demonstrate that re-instating gata4 is capable of recovering *sox7* —and to some extent *sox18—* expression. Mammalian *Sox7* expression also requires *Gata4* function in a mouse ESC model of cardiomyogenesis, where Gata4 is also uniquely capable of promptly inducing *Sox7* expression (Fig. 6). The discovery that this SoxF gene is also downstream of *Gata4* in our mouse ES cell model strongly validates our Xenopus data and furthermore demonstrates that molecular pathways involved in early vertebrate cardiomyogenesis tend to be conserved. We conclude that we have identified an important gene regulatory axis from *gata4* to SoxF family of transcription factors in heart muscle differentiation.

This gata4-SoxF regulatory axis is, however, only part of a much wider gata gene-mediated GRN for cardiomyogenesis. Future experiments will be required to explore other *gata4*-regulated candidate genes identified by our transcriptomics analysis (and those regulated by *gata5* and *gata6*, see Fig. 1; and Table 1 in Afouda et al. (2017)), and to determine whether the transcriptional regulation of *sox7* and *sox18* by GATA4 is direct or involves important yet unidentified intermediary factors. It is expected that *gata4* function in cardiomyogenesis involves other downstream mediators, since the *sox7* and *sox18* knockdown phenotype is less severe then the *gata4* knockdown (Fig. 2) and also since experimentally reinstating sox7 and sox18 function cannot fully recover cardiomyogenesis in a *gata4* knockdown (Fig. 4). Identification of such key genes downstream of *gata4* and of other cardiogenic gata genes promises to provide a better understanding of human cardiomyopathies resulting from *GATA4* mutations (Garg et al., 2003, Rajagopal et al., 2007) and mouse phenotypes such as acardia in *Gata4* and *Gata6* double mutant embryos (Zhao et al., 2008).

An important role for SoxF genes for cardiovascular development is further supported by the cardiovascular failure of mice lacking *Sox7* that leads to death of embryos at embryonic day 10.5 (Wat et al., 2012), by the importance of *Sox7* in arterial specification (Hermkens et al., 2015) and by the essential functions of *sox7* and *sox18* in Xenopus cardiomyogenesis (Zhang et al., 2005b). Our data indicates possible redundancy between *sox7* and *sox18* as knockdown of both together leads to a more severe reduction of cardiac marker gene expression than either of them alone (Fig. 3) (see also Zhang et al., 2005b).

**Fig. 7:**
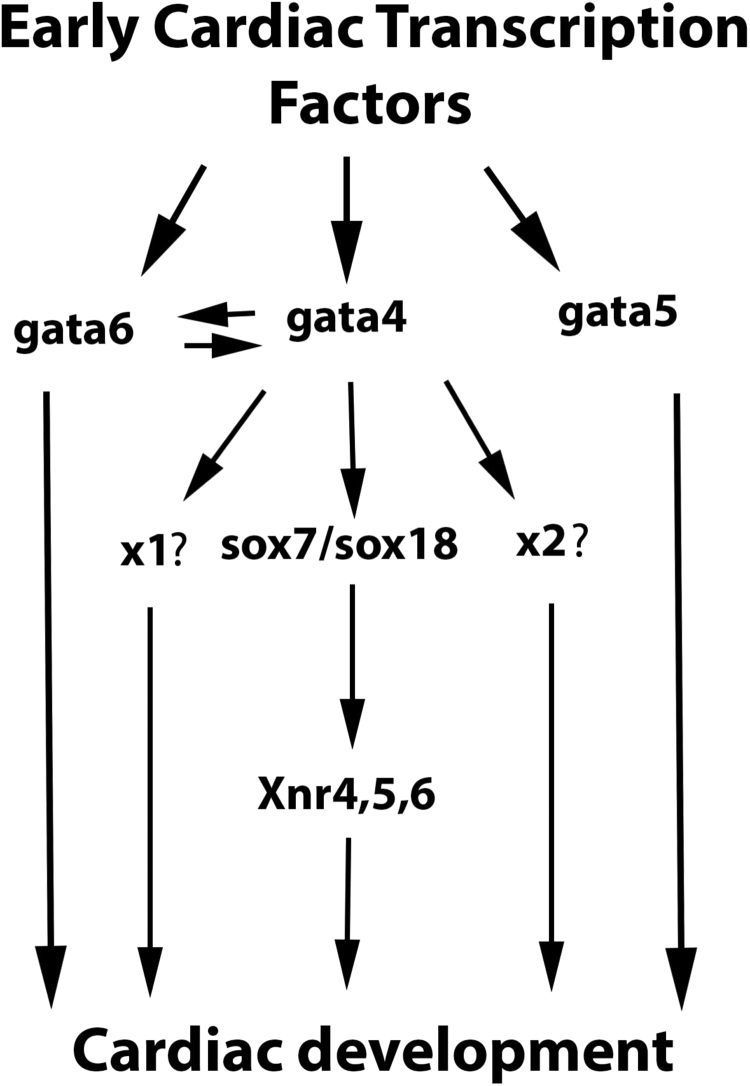
Proposed model to depict the functional differences between cardiogenic gata paralogs during cardiomyogenesis. The SoxF paralogs sox7 and sox18 mediate gata4 but not gata5 and gata6 function during cardiomyogenesis. All cardiogenic gata function after induction of early cardiac transcription factors where sox7 and sox18 act downstream to mediate only some of gata4 but not gata5 and gata6 function during cardiomyogenesis (for details, see text in Discussion Sections). ×1? and ×2? indicate yet unidentified intermediary factors.

However, there are also SoxF-independent regulatory mechanisms expected to drive cardiomyogenesis. It is intriguing that although gata5 and gata6 are not able to reinstate *sox7* or *sox18* expression in a *gata4* knockdown, they can substantially reinstate expression of cardiac differentiation markers (Fig. 4). This suggests *sox7*- and *sox18*-independent pathways downstream of *gata5* and *gata6* (Fig. 7). This confirms our and other previous studies (Afouda and Hoppler, 2011, Ieda et al., 2010) uncovering divergent functional roles of cardiogenic GATA factors during cardiomyogenesis. Our discovery of *sox7* and *sox18* as genes specifically regulated by *gata4* not only increases our general understanding of cardiogenesis but also provides specifically insights into the gene regulatory mechanisms involved. Functional interactions with other regulators of cardiogenesis are likely, for instance, SOX proteins have previously been shown to interact with β-catenin (Kormish et al., 2010, Zorn et al., 1999), which can function as an inhibitor during early cardiogenesis (Afouda et al., 2008, Ueno et al., 2007). However, further studies have shown that while SOX7 physically interacts with β-catenin and functionally inhibits Wnt signalling pathway activity; a version of SOX7 that cannot interact with β-catenin is still able to induce cardiogenesis (Zhang et al., 2005b) suggesting that this aspect of Sox7 function in cardiogenesis is Wnt/β-catenin-independent. Since *sox7* and *sox18* have been reported to induce the expression of *nodal4*, *nodal5* and *nodal6* in cardiogenesis in Xenopus explants (Zhang et al., 2005a, Zhang et al., 2005b) we include them in a model to depict the functional differences between gata paralogs during cardiomyogenesis (Fig. 7).

It is also the wider embryonic context with neighbouring tissues that provides further dependability and built-in redundancy to the gene regulatory mechanisms driving cardiomyogenesis. While studying cardiogenesis in relative isolation in Activin-induced cardiac explants was effective for uncovering the gata4-SoxF regulatory axis (Fig. 3), the wider embryonic context of DMZ explants suggests that there are additional pathways allowing all cardiogenic gata factors to relieve inhibition of cardiac differentiation in the *sox7* knockdown (Fig. 5A, B). Future experiments will need to explore any role particularly for the endoderm: it is intriguing for instance that we observe a further loss of expression of cardiomyocyte differentiation markers in the *sox7* knockdown (Fig. 3H, I), and of earlier cardiac progenitor markers such as *mespa* (data not shown), when cardiogenic GATA function is experimentally activated during early stages, which could be explained by the pro-endoderm-inducing activity of these GATA factors (Afouda et al., 2005). In support, we do observe a slight increase in the induction of the third SoxF paralog sox17α, a known endoderm marker (data not shown). A role of the endoderm for cardiomyogenesis in the wider embryonic context is of course consistent with the previously proposed requirements of signals from this layer for cardiac development (Foley et al., 2007, Foley and Mercola, 2005, Liu et al., 2014, Nascone and Mercola, 1995, Nascone and Mercola, 1996, Schneider and Mercola, 2001).

In conclusion, our results identify an important gene regulatory axis from *gata4* to the SoxF paralogs *sox7* and *sox18* for heart muscle differentiation, which is conserved in wider embryonic contexts (DMZ and entire embryo, Fig. 5) and in mammalian cardiomyogenesis (Fig. 6). Our findings represent a further important advance in the molecular dissection of the regulatory mechanisms controlling heart formation. They pave the way for further investigations into elucidating GRNs that are involved downstream of *gata4* and the other paralogs *gata5* and *gata6* in heart muscle development.

## 4 Materials and methods

### 4.1 Ethics statement

All Xenopus experiments were performed according to the University of Aberdeen's Code of Practice on the Use of Animals in Research as well as the legal requirements of the Animals (Scientific Procedures) Act 1986 (Licence PPL 60/4376) and the Home Office Code of Practice guidance.

### 4.2 Expression constructs, mRNA synthesis and morpholinos

Activin β B, gata4GR, gata5GR and gata6GR DNA constructs for mRNA synthesis have been described previously (Afouda et al., 2005, Afouda et al., 2008, Afouda and Hoppler, 2011). *Xenopus laevis* sox18GR DNA construct was made by in frame fusion of its coding region to the region encoding the hormone-inducible domain of human gluco-corticoid receptor in pSP64T-GR as previously described (Tada et al., 1997). To this end, a *BamHI* site was created at the end of the amplified coding sequence and inserted into *Bgl*II-digested vector. The following primer sequences were used to amplify *Xenopus laevis* sox18 (pDONR223Xsox18 a kind gift from Prof Aaron Zorn and Scott Rankin). Xsox18: 5′ GGA TCG GAT CCA CCA GGA TGC ATA GAT CTA GC 3′ for the forward primer and 5′ GAT CCG GAT CCC TAG CCA GTA ATA CAG GGG 3′ for the reverse primer (accession number NM_001088635). The *Xenopus laevis* sox7 fusion construct (Xsox7GR) was made from stage 32 cardiac explants cDNA synthesised according to a previously described protocol (Weber et al., 2000). To this end we’ve designed specific primers to sox7 sequence accession number NM_001085868 flanked by *Bgl*II sequence to amplify its cDNA. The following primers sequences were used: Xsox7: 5′ GGA TCA GAT CTA CCA GGA TGA CTA CCC TGA TGG GAT CC 3′ for the forward primer and 5′ GAT CCA GAT CTA GAA ACA CTA TAA CTG TTG 3′ for the reverse primer. The PCR product was *Bgl*II-digested and inserted in frame into pSP64T-GR vector as above. All the constructs were checked by restriction digestion and by sequencing. All fusion plasmids were *Sal*I-linearized and in vitro transcribed with SP6 using mMESSAGE mMACHINE kits (Ambion) according to the manufacturer's instruction. The following amounts of RNA were injected: 50 fg for Activin, 200–1000 pg for all other constructs per embryo (see Figures legends). Xenopus gata4 splice morpholino, gata5 splice morpholino (Haworth et al., 2008), gata6 morpholino (Peterkin et al., 2003), Xsox7 and Xsox18 (Zhang et al., 2005b) morpholinos have been previously described. The amounts of MOs injected per embryo are: 50 ng (gata4, 4MO), 8 ng (gata5, 5MO), 10 ng (gata6, 6MO), 30 ng for single (sox7, 7MO) and (sox18, 18MO) and 15 ng of each when combined.

### 4.3 Embryos and explants culture

*Xenopus laevis* embryos were obtained as previously described (Afouda et al., 2005). Embryos and explants culture as well as embryos injection were as described in Afouda et al. (2008) and Afouda and Hoppler (2011). Animal cap explants were excised as previously described (Afouda, 2012) and where applicable dexamethasone was added at final concentration of 10 µM (at either stage 8 or 13, see Figures legends) for activation of GR-fusion proteins. Live GFP beating explants imaging was performed using ZEISS Axio Observer Z.1 microscope with the Axiovision software and movies were taken using Leica M60 microscope mounted with Leica MC 170 HD camera. Transgenic embryos were obtained from myl2-GFP lines. They were sourced from National Xenopus Resource (at Marine Biology Laboratory, Woods Hole, Massachusetts, USA) and were previously generated (Latinkic et al., 2004).

### 4.4 RNA extraction, RNA expression analysis and protein analysis

Whole mount in situ hybridisation (WISH) was conducted as previously described (Ciau-Uitz et al., 2000). A digoxigenin-labelled RNA probe was prepared with T7 polymerase, using High Yield Megascript Kit (Ambion) from *Not*I-linearized plasmid template for tnni3 (Drysdale et al., 1994). The abundance of RNAs was determined using semi-quantitative Reverse Transcriptase PCR (RT-PCR) (Afouda and Hoppler, 2011) and real time quantitative Taq man assays using the comparative CT method. For this, at least three repeats were done for each experimental group and 20–30 cardiogenic explants were analysed for each condition (or 25–50 mESC Embryoid Bodies). For each experimental repeat measurements for each condition were conducted in triplicate and relative mRNA expression levels were calculated by normalisation to Xenopus *odc* or mouse *Gapdh* mRNAs. Raw data were analysed with the ΔΔCT method (Livak and Schmittgen, 2001). Data are mean±SEM, n = 3, P ≤ 0.05. Data presented are representative of one experiment. Reactions were run on Abi 7700 Sequence Detector (Afouda et al., 2005, Peterkin et al., 2003). Sequences of primers and probes for quantitative Taq man were as described in (Peterkin et al., 2003). Sequence of primers used for semi-quantitative RT-PCR are as follow: gata4, myl2, tnni3 and odc (Afouda and Hoppler, 2011); *Xenopus laevis* mespa and mespb (Hitachi et al., 2009); *Xenopus laevis* Isl1 (Gessert and Kühl, 2009); sox17α (Afouda et al., 2005). For both sox7 and sox18 the primers used were designed based on accession numbers mentioned above that are homeologs of the ones identified in our RNA-seq results and are as follow: sox7 primers 5′ ATG ACT ACC CTG ATG GGA TCC TAC AGC 3′ for forward primer and 5′ AGA AAC ACT ATA ACT GTT GTA GTA CG 3′ for reverse primer and for sox18 5′ ATG CAT AGA TCT AGC TAC TGC AGA G 3′ for forward primer and 5′ GCC AGT AAT ACA GGG GGT ATA GTA C 3′ for reverse primer. Protein extraction and western blot analysis were as previously described (Afouda et al., 2005).

RNAs were extracted from ES cells and Embryoid Bodies using the Absolutely RNA Microprep Kit (Agilent) according to the manufacturer's instructions. cDNAs were prepared as previously described (Afouda et al., 2005) and quantitative PCR was performed using LightCycler 480 Probes Master Mix from Roche with Universal Probe Library (UPL) and reaction run on LightCycler 480 machine.

### 4.5 RNA-seq experiments and analysis

At least 30 explants were used for RNA preparation with a previously described protocol (Afouda and Hoppler, 2011, Afouda et al., 2008). RNA quantity and quality were checked on electrophoretic agarose gel, a fraction of which was used for validation with gene expression analysis by quantitative RT-PCR (Afouda et al., 2005) to confirm expected increase or decrease of known control gene expression before RNA-seq sequencing. RNA was isolated from three independent biological replicates for each condition. Illumina TruSeq RNA libraries were constructed and sequenced on the Illumina HiSeq_2500 platform at the Earlham Institute, Norwich Research Park, Norwich, UK. 100 bp paired-end sequencing reads were aligned to the *Xenopus laevis* genome (version 9.1) using HiSat2 (Kim et al., 2015) and quantification was done using featureCounts (Liao et al., 2014). Differential expression analysis was performed using DESeq. 2 (Love et al., 2014) with an adjusted p value<0.05. Differentially expressed genes were identified using a threshold of log-2 fold change>1 (for at least two times increased) or<−1 (for at least two times reduced) in comparison to Activin-induced Xenopus animal cap cardiac explant controls. Analyses of differentially expressed genes were performed using Partek genomics Suite 6.6. Similarly hierarchal clustering was performed in Partek Genomic genomics Suite 6.6. Counts were averaged for each condition and then these averages were standardised to have the same mean across the different conditions. Similarity between each gene expression profile within each cluster was then computed with Euclidean distance and shown as heatmap (Fig. 1G, I). For gene ontology (GO) analyses, GO classes containing at least six genes were taken into consideration.

### 4.6 ES cell culture and differentiation

Inducible reporter Mesp1/Gata4 and iGata cell lines were obtained from Professors Cedric Blanpain (Université Libre de Bruxelles, Belgium, (Bondue and Blanpain, 2010)) and Todd Evans (Weill Cornell Medical College, USA, (Holtzinger et al., 2010; Turbendian et al., 2013)), respectively. Inducible Mesp1 cells were maintained and differentiated as previously described (Bondue and Blanpain, 2010, Bondue et al., 2008). The iGata4/5/6 cells were maintained and differentiated as previously described (Holtzinger et al., 2010, Turbendian et al., 2013), except that Embryoid Bodies were differentiated in hanging drop culture. shRNAs were obtained from Qiagen and stable transfected cell lines were generated according to manufacturer's instructions.

## Appendix A Supplementary material

Supplementary material Fig. S1 *sox7 and sox18 mediate gata4 but not gata5 or gata6 function during cardiomyogenesis.* (A) RT-PCR analysis of endogenous gata4 and gata5 mRNAs of explants injected with morpholinos for either gata4 (4MO), gata5 (5MO) or gata6 (6MO). 4MO and 5MO cause splicing out of exon 4 in gata4 and gata5 mRNAs, respectively. 3-4-5, cDNA that contains exon 4 and parts of exons 3 and 5; 3-5, cDNA without exon 4. (B and C) List of genes from Fig. 1G and I (respectively) in the specifically gata4-regulated subclusters 1 and 2 containing sox18 and sox7, respectively. (D) Proteins production of exogenous over-expressed dexamethasone-inducible cardiogenic GATA in explants in the presence of either control, sox7/18 or both sox7 and 18 morpholinos (MOs). Note that the presence of MOs did not affect expression or stability of the exogenous over-expressed protein and the observed reduced protein level in the presence of dexamethasone that allows now freed HSP90 bounded protein to translocate into the nucleus. (E) Morpholino-mediated inhibition of sox7 or sox18 or both causes reduction in number of explants differentiating into beating cardiomyocytes tissue as presented in suppl movie 2. (F) Protein production of exogenous over-expressed dexamethasone-inducible cardiogenic gata in explants in the presence of sox7 MO. Note the same observations mentioned above in D. (G) Protein production of over-expressed dexamethasone-inducible sox7/18 in Activin-induced cardiac explants in the presence of either control or gata 4 MOs. Again note the same observations mentioned above in panels D and F. Erk2 represents loading control of Mitogen-activated protein kinase (MAPK) family. Dexamethasone was added at control stage 8. Other abbreviations are same as in previous figures legends.
