# Supplementary figures and images for: Genome-wide transcriptomics analysis identifies sox7 and sox18 as specifically regulated by gata4 in cardiomyogenesis
Source: Dev Biol. 2018 Feb 1;434(1):108–20. doi: 10.1016/j.ydbio.2017.11.017 (PMC5814753; doi:10.1016/j.ydbio.2017.11.017)

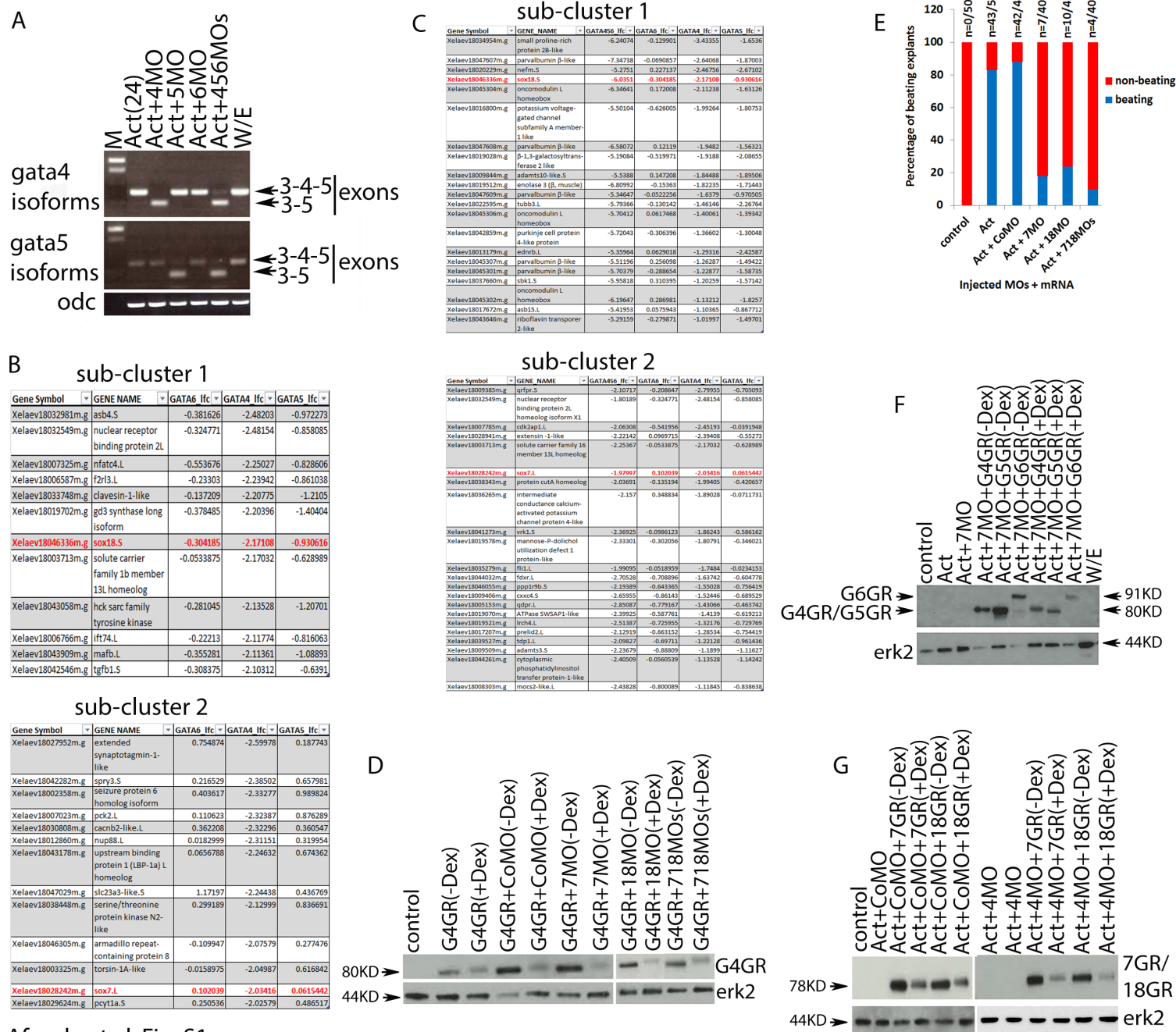

Afouda et al. Fig. S1

Supplement: Supplementary file 8 — Supplementary material Fig. S1 sox7 and sox18 mediate gata4 but not gata5 or gata6 function during cardiomyogenesis. (A) RT-PCR analysis of endogenous gata4 and gata5 mRNAs of explants injected with morpholinos for either gata4 (4MO), gata5 (5MO) or gata6 (6MO). 4MO and 5MO cause splicing out of exon 4 in gata4 and gata5 mRNAs, respectively. 3-4-5, cDNA that contains exon 4 and parts of exons 3 and 5; 3-5, cDNA without exon 4. (B and C) List of genes from Fig. 1G and I (respectively) in the specifically gata4-regulated subclusters 1 and 2 containing sox18 and sox7, respectively. (D) Proteins production of exogenous over-expressed dexamethasone-inducible cardiogenic GATA in explants in the presence of either control, sox7/18 or both sox7 and 18 morpholinos (MOs). Note that the presence of MOs did not affect expression or stability of the exogenous over-expressed protein and the observed reduced protein level in the presence of dexamethasone that allows now freed HSP90 bounded protein to translocate into the nucleus. (E) Morpholino-mediated inhibition of sox7 or sox18 or both causes reduction in number of explants differentiating into beating cardiomyocytes tissue as presented in suppl movie 2. (F) Protein production of exogenous over-expressed dexamethasone-inducible cardiogenic gata in explants in the presence of sox7 MO. Note the same observations mentioned above in D. (G) Protein production of over-expressed dexamethasone-inducible sox7/18 in Activin-induced cardiac explants in the presence of either control or gata 4 MOs. Again note the same observations mentioned above in panels D and F. Erk2 represents loading control of Mitogen-activated protein kinase (MAPK) family. Dexamethasone was added at control stage 8. Other abbreviations are same as in previous figures legends. [file mmc1.pdf]
